# Urinary tract infections at an Australian sexual health service: bacterial etiologies, antibiotic susceptibilities, and antimicrobial prescribing patterns

**DOI:** 10.1128/spectrum.02674-25

**Published:** 2026-05-05

**Authors:** S. E. Carter, E. L. Plummer, L. A. Vodstrcil, V. De Petra, I. Abbott, N. Sherry, C. K. Fairley, C. S. Bradshaw

**Affiliations:** 1 School of Translational Medicine, Monash University 161666, Melbourne, Victoria, Australia; 2 Melbourne Sexual Health Centre, Alfred Health 198098, Carlton, Victoria, Australia; 3 Centre for Epidemiology and Biostatistics, Melbourne School of Population and Global Health, The University of Melbourne, Melbourne, Victoria, Australia; 4 Microbiological Diagnostic Unit Public Health Laboratory, Department of Microbiology and Immunology, University of Melbourne, at the Peter Doherty Institute for Infection and Immunity, Melbourne, Victoria; 5 Department of Infectious Diseases and Microbiology Unit, Alfred Hospital and School of Translational Medicine, Monash University 2541, Melbourne, Victoria, Australia; 6 Department of Infectious Diseases & Immunology, Austin Health 3805, Heidelberg, Victoria, Australia; University of Saskatchewan, Saskatoon, Canada

**Keywords:** urinary tract infection, antimicrobial agents, antibiotic resistance

## Abstract

Urinary tract infections (UTIs) are one of the most common infections. Management of UTIs often involves empiric antibiotics, which can contribute to antibiotic overuse. We aimed to investigate the causative organisms, prevalence of antimicrobial resistance, and empiric prescribing practices to improve antibiotic use for UTIs. We conducted a retrospective audit of UTIs in premenopausal nonpregnant persons with a vagina aged 18–51 years attending the Melbourne Sexual Health Centre from 2 January 2018 to 24 January 2023. We calculated the proportion of culture samples with pathogen(s) isolated, and their antimicrobial resistance profiles with 95% confidence intervals (CIs). We assessed adherence to first-line Australian Therapeutic guideline recommendations for acute uncomplicated lower UTIs (trimethoprim, nitrofurantoin, and cefalexin) during our study period. We included 1,680 acute UTI episodes (*n* = 1,569 patients). Midstream urine culture was performed in 1,503 episodes, with 911 (60.6%, 95% CI: 58.1–63.1) classified as laboratory-defined UTIs. The most common isolate was *Escherichia coli* (*n* = 615/948; 64.9%, 95% CI: 61.7–67.9). Of the first-line Australian Therapeutic Guideline recommended antibiotics, *E. coli* isolates were most commonly resistant to trimethoprim (*n* = 195/612; 31.9%, 95% CI: 28.2–35.7). Empiric antibiotics were prescribed for 1,533 (91.3%, 95% CI: 89.8–92.6) episodes. Most prescriptions (*n* = 1,465/1,533; 95.6%, 95% CI: 94.4–96.5) adhered to first-line recommendations, and trimethoprim accounted for 842 (54.9%, 95% CI: 52.4–57.4) prescriptions. At Australia’s largest sexual health service, *E. coli* caused two-thirds of UTIs, with one-third of *E. coli* isolates demonstrating trimethoprim resistance. Empiric antibiotics were commonly prescribed and compliant with national recommendations; trimethoprim accounted for >50% of empiric prescriptions. Trimethoprim resistance indicates changes to empiric recommendations could be needed.

**IMPORTANCE:**

*Escherichia coli* was the most common uropathogen isolated in women with uncomplicated urinary tract infection (UTI) over the past 5 years. One-third of these infections were resistant to trimethoprim, an antibiotic recommended as first-line presumptive therapy for UTIs in Australia and prescribed in over 50% of these *E. coli* urinary tract infections. These data demonstrate higher than previously reported levels of trimethoprim resistance in community-acquired *E. coli* UTIs in Australia, and the need for changes to empiric antimicrobial recommendations.

## INTRODUCTION

Urinary tract infections (UTIs) are one of the most common community-acquired and nosocomial infections, affecting >400 million people globally each year (1–3). In Australia, UTIs represent 1.2% of all presenting problems managed during general practice consultations (4). Individuals with female urogenital anatomy are disproportionately affected; up to 50% of females experience a UTI during their lifetime, and the incidence of uncomplicated UTIs is highest among young, sexually active females (5–7).

UTIs are a leading indication for antibiotics, and empiric treatment is the current mainstay of UTI management throughout much of the world (8). During the study period, the Australian Therapeutic Guidelines recommended trimethoprim and nitrofurantoin as first-line antibiotics for the empiric management of acute, uncomplicated lower UTIs in nonpregnant women. If neither trimethoprim or nitrofurantoin could be used, cefalexin was recommended. The Australian Therapeutic Guidelines were updated in 2025, and fosfomycin is now a first-line antibiotic option, in addition to trimethoprim, nitrofurantoin, and cefalexin. Notably, the Australian Therapeutic Guidelines in 2025 introduced a first-line antibiotic sparing approach, whereby a trial of nonantibiotic therapy with ibuprofen to manage symptoms can be considered for nonpregnant adult females younger than 65 years with mild symptoms of acute cystitis who are not immunocompromised (9). Importantly, antimicrobial resistance (AMR) among uropathogens is rising, with significant detrimental impacts on the effectiveness of empiric UTI therapies (10, 11). Choice of empiric antibiotic therapy should be based on an individual patient’s risk profile, risk-benefit assessment of drug toxicity and spectrum of cover, population studies, and antibiogram data. Without this timely information, empiric management may promote inappropriate antibiotic use and fuel the development of AMR (12).

In Australia, the prevalence of AMR in uropathogenic *Escherichia coli* is rising, although there are limited data from community or sexual health clinics (13, 14). Isolates with AMR, particularly those exhibiting resistance to third-generation cephalosporin antibiotics (e.g., ceftriaxone), have been highlighted by the World Health Organization as a “critical priority” for the research and development of new antibiotics (15, 16). Lower rates of antimicrobial resistance amongst Gram-positive uropathogens like *Staphylococcus saprophyticus* have been reported in Australia (10, 12).

For patients to be appropriately managed, their diagnosis must be correct; however, there is no perfect diagnostic test or universally agreed diagnostic criteria for lower UTIs (17–20). Ultimately, the goal when treating uncomplicated lower UTIs is to manage symptoms and optimize antibiotic stewardship while ensuring minimal undesired effects, including complications like ascending infection, and collateral damage (i.e., the ecological effects of antimicrobials on the microbiota) (21, 22).

As resistance patterns vary with geography, region-specific data are important to inform local management (6). Therefore, this study aimed to improve understanding of antimicrobial resistance in uropathogens and assess the appropriateness of prescribing practices and guideline recommendations in Australia’s largest sexual health service.

## RESULTS

### Description of study population

**Fig 1:**
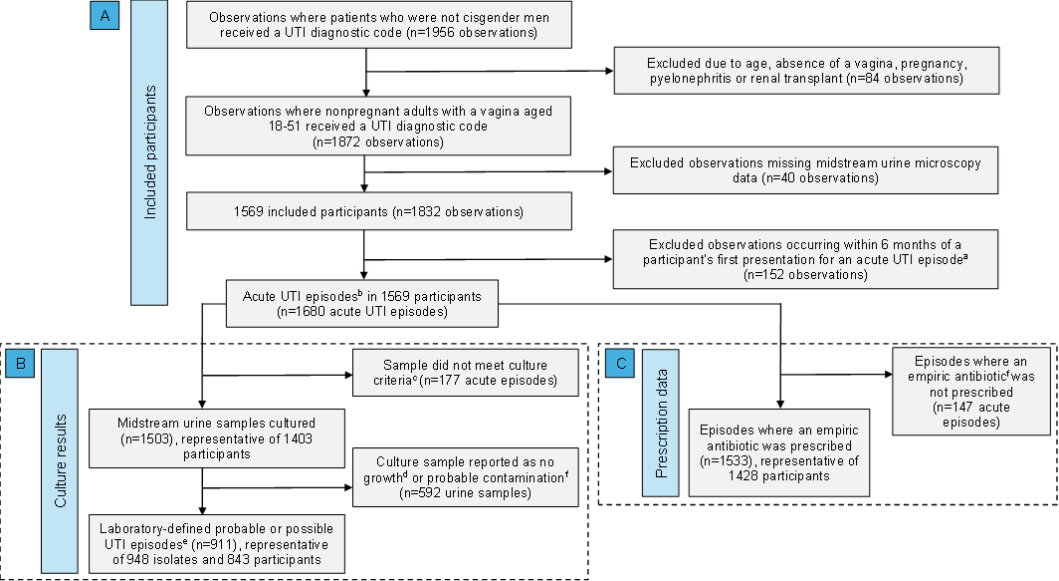
Population flowchart detailing inclusion in analysis using data from patients who received a clinician diagnosis of a urinary tract infection at the Melbourne Sexual Health Centre between January 2018 and January 2023. Panel A relates to demographic information, and urine dipstick and microscopy findings. Boxes indicate panels B and C, which represent populations that are not mutually exclusive. Panel B depicts the available urine culture data, and panel C represents the included empiric prescription data. UTI, urinary tract infection. ^a^for participants with multiple visits, only the first visit in the same 6-month period was included to prevent repeat presentations for the same UTI episode influencing estimates. ^b^Acute UTI episodes are defined as the first included presentation per person per 6 months during our study period. ^c^While all included acute episodes had midstream urine samples sent for culture, samples must meet at least one of the following criteria for culture to be performed: positive nitrites or leukocyte esterase on urine dipstick, >10 × 10^6^ polymorphonuclear cells/L on microscopy, or clinician request. ^d^Growth <10^3^ CFU/mL is reported by the laboratory as no growth. ^e^See supplemental material for full criteria for laboratory classification of samples as “probable contamination,” “possible UTI,” and “probable UTI.” ^f^Antibiotics prescribed before urine culture and susceptibility results were available were classified as empiric.

During the study period, there were 1,956 UTI diagnoses among cisgender women, trans men, and non-binary patients attending Melbourne Sexual Health Centre (MSHC) (Fig. 1A). We excluded 84 (*n* = 84/1956; 4.3%, 95% CI: 3.4–5.3) observations due to pregnancy, absence of a vagina, age, pyelonephritis, or renal transplant. An additional 40 (*n* = 40/1872; 2.1%, 95% CI: 1.5–2.8) observations were excluded due to missing midstream urine dipstick and/or microscopy, culture, and sensitivity (MCS) results. Among these excluded observations, there were 20 (*n* = 20/1,872; 1.1%, 95% CI: 0.7%–1.6%) in which a diagnosis of a UTI was given without available urine dipstick and MCS results. Of the remaining 1,832 observations, 152 (8.3%, 95% CI: 7.1%–9.7%) occurred less than 6 months after an eligible acute UTI episode (defined in “Materials and Methods” section) and were excluded. Thus, 1,680 acute UTI episodes among 1,569 persons with female anatomy were included in the analyses; most (99%) identified as female (Table 1). The median number of episodes per person was one and ranged from one to four. The median age of participants was 26 years with an interquartile range of 23–31 years. Most participants (*n* = 1,156/1,569; 73.7%, 95% CI: 71.4%–75.8%) reported ≥1 sexual partner in the 3 months prior to their first presentation during the study period (Table 1).

**TABLE 1:** Demographic characteristics and sexual practices of included participants at the time of their first clinician diagnosis of a urinary tract infection at the Melbourne Sexual Health Centre between January 2018 and January 2023^*b*^

	Number (%): *n* = 1,569
Age	
Median (IQR)	26.3 (23.2–30.6)
Gender	
Female	1,552 (98.9)
Trans male	13 (0.8)
Non-binary	4 (0.3)
Sexual practices in the last 3 months	
Number of sexual partners (any gender)	
1	406 (25.9)
>1	750 (47.8)
Unknown	413 (26.3)
Male condom use	
Always	160 (10.2)
Sometimes	688 (43.8)
Never	277 (17.7)
Unknown, declined to answer, or not applicable	444 (28.3)
Contraception (excluding condoms)	
Hormonal contraception or IUD	537 (34.2)
Other contraception^*a*^	615 (39.2)
Unknown, declined to answer, or not applicable	417 (26.6)
Current sex worker	
Yes	128 (8.2)
No	1,121 (71.4)
Unknown or declined to answer	320 (20.4)

^*a*^ Includes withdrawal, rhythm or calendar, or diaphragm.

^*b*^ IQR, interquartile range; IUD, intrauterine device.

Of the included participants, two (*n* = 2/1,569; 0.1%) were living with human immunodeficiency virus (HIV). *Chlamydia trachomatis* was detected in 100 (*n* = 100/1,680; 6.0%) included episodes, *Neisseria gonorrhoeae* was detected in 33 (*n* = 33/1,680; 2.0%) episodes, and *Mycoplasma genitalium* was detected in 27 (*n* = 27/1,680; 1.6%) episodes (Table SA1).

### Urinary investigation results

Midstream urine dipstick results from all included UTI episodes (*n* = 1,680) are presented in Table 2. Of these, 1406 samples (83.7%, 95% CI: 81.8–85.4) were positive for leukocyte esterase, and 237 (14.1%, 95% CI: 12.5–15.9) were positive for nitrites. Of the 1,680 samples sent for urine microscopy, 1,445 (86.0%, 95% CI: 84.3–87.6) demonstrated significant levels of polymorphonuclear cells, and 1,617 (96.3%, 95% CI: 95.3–97.2) had significant numbers of bacteria (Table 2).

**TABLE 2:** Midstream urine dipstick and microscopy results for each acute episode of a clinician-diagnosed urinary tract infection^*b*^

Urine dipstick finding	*n*, % [95% CI] *n* = 1,680
Leukocyte esterase	
Negative	269, 16.0 [14.3–17.9]
15 (trace)	278, 16.5 [14.7–18.4]
70+	333, 19.8 [17.9–21.8]
125++	510, 30.4 [28.2–32.7]
500+++	285, 17.0 [15.3–18.9]
No data^*a*^	5, 0.3 [0.1–0.7]
Nitrites	
Negative	1,436, 85.5 [83.7–87.1]
Positive	237, 14.1 [12.5–15.9]
No data^*a*^	7, 0.4 [0.2–0.9]
Microscopy finding	
Polymorphonuclear cells (cells ×10^6^/L)	
<10	234, 13.9 [12.3–15.7]
10–200	775, 46.1 [43.7–48.5]
>200	670, 39.9 [37.5–42.3]
No data^*a*^	1, 0.1 [0.0–0.3]
Erythrocytes (cells ×10^6^/L)	
0	4, 0.2 [0.1–0.6]
<10	794, 47.3 [44.9–49.7]
10-200	593, 35.3 [33.0–37.6]
>200	288, 17.1 [15.3–19.0]
No data^*a*^	1, 0.1 [0.0–0.3]
Bacteria (bacterial cell/10 small grids)	
None	58, 3.5 [2.7–4.5]
No significant (<1)	1, 0.1 [0.0–0.3]
Light (1–3)	1,100, 65.5 [63.1–67.8]
Moderate (3–15)	306, 18.2 [16.4–20.1]
Heavy or very heavy (>15)	211, 12.6 [11.1–14.3]
No data^*a*^	4, 0.2 [0.1–0.6]
Epithelial cells	
None present	220, 13.1 [11.5–14.8]
Any present (≥1)	1,458, 86.8 [85.1–88.4]
No data^*a*^	2, 0.1 [0.0–0.4]

^*a*^ Some aspects of urine microscopy data were not reported for up to seven acute episodes, e.g., due to a blood-stained sample.

^*b*^ CI, confidence interval.

**Fig 2:**
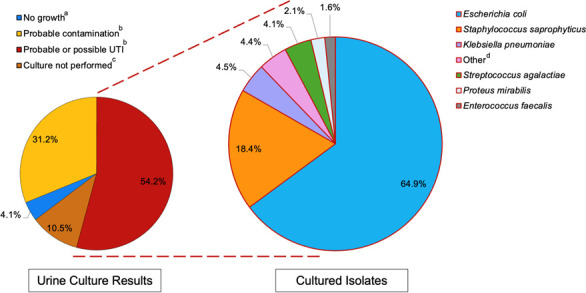
Midstream urine culture results for each acute episode of a clinician-diagnosed urinary tract infection (*n* = 1,680 episodes; *n* = 1,569 participants), and cultured isolates from each laboratory-defined urinary tract infection sample (*n* = 911 acute episodes; *n* = 948 isolates; *n* = 843 participants). UTI, urinary tract infection. ^a^Growth <10^3^ CFU/mL is reported by the laboratory as no growth. ^b^See Supporting Information for full criteria for laboratory classification of samples as “probable contamination,” “possible UTI,” and “probable UTI.” ^c^While all included acute episodes had midstream urine samples sent for culture, samples must meet at least one of the following criteria for culture to be performed: positive nitrites or leukocyte esterase on urine dipstick, >10 × 10^6^ polymorphonuclear cells/L on microscopy, or clinician request. “Other” includes uropathogens cultured in <1% of included samples (full list of organisms provided in Table SA2).

Of the 1,680 samples sent for MCS, 1,503 (89.5%, 95% CI: 88.0–91.0) met the criteria for culture, and 911 (60.6%, 95% CI: 58.1–63.1) were defined by the laboratory as a “probable UTI” or “possible UTI,” which required at least 10^3^ CFU/mL of growth on culture (see Supporting Information for laboratory classification criteria) (Fig. 1B and 2). From these 911 samples, 948 bacterial isolates were cultured; 875 (*n* = 875/911; 96.0%, 95% CI: 94.6–97.2) episodes had significant growth of one isolate, 35 (*n* = 35/911; 3.8%, 95% CI: 2.7–5.3) had potentially significant growth of two isolates, and one (*n* = 1/911; 0.1%, 95% CI: 0.0–0.6) had potentially significant growth of three isolates.

The most frequent isolates cultured were *E. coli* (*n* = 615/948; 64.9%, 95% CI: 61.7–67.9), *S. saprophyticus* (*n* = 174/948; 18.4%, 95% CI: 15.9–21.0), and *K. pneumoniae* (*n* = 43/948; 4.5%, 95% CI: 3.3–6.1) (Fig. 2). A list of cultured isolates is provided in Table SA2.

Table 3 shows the antibiotic susceptibility profiles of individual uropathogens where the total number of an isolated species was ≥30. *E. coli* demonstrated most resistance to trimethoprim, with susceptibility in only 68.1% (*n* = 417/612; 95% CI: 64.3–71.8). A high proportion of *K. pneumoniae* isolates were susceptible to trimethoprim and cefalexin (≥90.7%). For the two most common Gram-positive isolates (*S. saprophyticus* and *Streptococcus agalactiae* [Group B Streptococcus]), all isolates were susceptible to nitrofurantoin and cefalexin. There was no trimethoprim susceptibility data available for *S. saprophyticus* or *S. agalactiae*; of note, trimethoprim is not recommended for treatment of *S. agalactiae*. There was no susceptibility data for fosfomycin, which was not a first-line antibiotic recommendation during our study period.

**TABLE 3:** Antimicrobial susceptibility profiles for common uropathogens in laboratory-defined urinary tract infections, displayed as percentage (*n* = 871)^*a*^

	Number of isolates	Trimethoprim	Nitrofurantoin	Cefalexin	Amoxicillin^*e*^	Amoxicillin and clavulanic acid	Trimethoprim and sulfamethoxazole	Norfloxacin	Ciprofloxacin	Fosfomycin	Ceftriaxone
**Gram-negative isolates**											
*Escherichia coli*	615	68.1^*b*^ *n* = 612	97.2 *n* = 614	89.8 *n* = 615	53.2^*c*^ *n* = 357	89.1 *n* = 612	72.3 *n* = 357	77.9 *n* = 358	88.2 *n* = 357	X	92.4 *n* = 357
*Klebsiella pneumoniae*	43	93.0 *n* = 43	X	90.7 *n* = 43	IR	95.3 *n* = 43	92.6^*d*^ *n* = 27	85.2^*d*^ *n* = 27	88.9^*d*^ *n* = 27	IR	88.9^*d*^ *n* = 27

	Number of isolates	Nitrofurantoin	Cefalexin	Trimethoprim and sulfamethoxazole							
**Gram-positive isolates**											
*Staphylococcus saprophyticus*	174	100.0 *n* = 174	92.0 *n* = 174	97.1 *n* = 174							
*Streptococcus agalactiae*	39	100.0 *n* = 36	100.0 *n* = 38	X							

^*a*^ Gray shading, >70% susceptible; X, not reported; IR, intrinsic resistance.

^*b*^ 60%–70% susceptible.

^*c*^ <60% susceptible.

^*d*^ *n* ≤ 30 isolates.

^*e*^ Ampicillin susceptibility results were used to predict amoxicillin susceptibility.

There were 27 (27/357; 7.6%, 95% CI: 5.0–10.8) third-generation cephalosporin-resistant *E. coli* isolates in laboratory-defined UTI samples. Of these, 20 (*n* = 20/27; 74.1%, 95% CI: 53.7–88.9) displayed resistance to trimethoprim, and 2 (*n* = 2/27; 7.4%, 95% CI: 0.9–24.3) were resistant to nitrofurantoin.

### Empiric antibiotic prescription results

Empiric antibiotics were prescribed in 1,533 (91.3%, 95% CI: 89.8–92.6) of the 1,680 included episodes (Fig. 1C; Fig. SA1). Of these empiric antibiotics, 842 prescriptions were for trimethoprim (54.9%, 95% CI: 52.4–57.4), and 586 were for cefalexin (38.2%, 95% CI: 35.8–40.7). Nitrofurantoin was the third least commonly prescribed first-line antibiotic (*n* = 37, 2.4%, 95% CI: 1.7–3.3). Fosfomycin was not prescribed during our study period. In total, 1,465 (95.6%, 95% CI: 94.4–96.5) prescriptions were in accordance with first-line Australian recommendations for acute uncomplicated lower UTIs in nonpregnant adult females (i.e., trimethoprim, nitrofurantoin, fosfomycin, or cefalexin).

**Fig 3:**
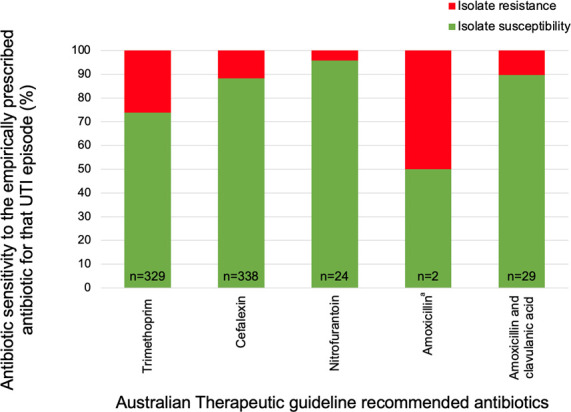
Clinician-diagnosed acute UTI episodes in which an appropriate antibiotic was prescribed based on the antimicrobial susceptibility profile of the isolate for that episode (*n* = 722). There is variability in the number of acute episodes where relevant antimicrobial susceptibility data are accessible as susceptibility data are only generated for laboratory-defined probable or possible urinary tract infections (see supplemental material for full criteria), and because the specific antibiotics that susceptibility and resistance are assessed against vary according to the isolate. ^a^Ampicillin susceptibility results were used to predict amoxicillin susceptibility. UTI, urinary tract infection.

We next assessed the proportion of episodes where the empiric antibiotic adequately covered the cultured isolate(s) based on the final susceptibility report (Fig. 3). There were 723 episodes where an empiric antibiotic was prescribed and susceptibility data for that antimicrobial were subsequently available (post prescription). Among these, 131 (18.1%, 95% CI: 15.4–21.1) episodes had an isolate(s) that was classified as resistant to the prescribed antibiotic. Of the episodes where empiric trimethoprim was prescribed, 86 had samples where at least one isolate demonstrated trimethoprim resistance (*n* = 86/329; 26.1%, 95% CI: 21.5–31.2).

## DISCUSSION

In this study of over 1,600 pre-menopausal nonpregnant persons with a vagina who received a clinician-diagnosis of a UTI, we found that *E. coli* was responsible for two-thirds of isolated uropathogens and *S. saprophyticus* accounted for nearly one in five isolates. Concerningly, almost a third of *E. coli* isolates were resistant to trimethoprim, which was the most prescribed empiric antibiotic in this clinic population. Empiric antibiotics were frequently prescribed, with an antibiotic dispensed before urine culture and antimicrobial susceptibility results were available in >90% of acute UTI episodes. Prescriptions almost always adhered to the previous and current Australian Therapeutic guideline first-line recommendations, with more than half of all prescriptions being for trimethoprim (9). In total, 18% of episodes had an isolate(s) subsequently found to be resistant to the prescribed empiric antibiotic for that episode, with most of these isolates demonstrating resistance to trimethoprim. Of those prescribed empiric trimethoprim, 26% of episodes had at least one uropathogen that was resistant to trimethoprim. Overall, we found a high proportion of UTIs were caused by well-recognized uropathogens for uncomplicated UTIs, and that *E. coli* resistance to trimethoprim was higher than national data but in line with local data from Melbourne (5, 23, 24).

A third of *E. coli* isolates were resistant to trimethoprim. This level of resistance is somewhat more prevalent than previously reported in Australia. A study of adult females diagnosed with uncomplicated acute cystitis attending general practices found 27% (*n* = 13/48) of urinary *E. coli* isolates were resistant to trimethoprim, although their sample size was small (24). In urine samples from 2009 to 2013, the proportion of community-acquired *E. coli* isolates with trimethoprim resistance was 21% in Australia (13). Additionally, a government report on antimicrobial use and resistance in Australia found that 22% of urinary *E. coli* samples from community settings were resistant to trimethoprim in 2020–2021. The same report demonstrated the proportion of hospital- and community-acquired *E. coli* with resistance to cefalexin and nitrofurantoin was 8% and 1%, respectively, in 2021. Resistance to amoxicillin in urinary *E. coli* isolates from community samples was reported in 41% of isolates (10). In our study, we found a high proportion of resistance to amoxicillin (53%) among *E. coli* isolated from urine samples. We also found slightly higher resistance for our most common isolates to cefalexin (<11%) and nitrofurantoin (<3%). Rates of third-generation cephalosporin-resistance in our *E. coli* isolates were similar (7.6%) to national data from community and hospital urine samples in 2021 (6.9%) (25). A major update to the latest Australian Therapeutic Guidelines was the introduction of a first-line antibiotic sparing approach, whereby a trial of nonantibiotic therapy for symptom management is suggested to be considered for nonpregnant adult females younger than 65 years with mild symptoms of acute cystitis who are not immunocompromised. Possible reduction in antibiotic prescribing rates as a result of this recommendation may slow development of antimicrobial resistance (9). There were no data on trimethoprim susceptibility for *S. saprophyticus* isolates as the VITEK 2 Gram-positive Susceptibility Card used for *Staphylococcus* species at our laboratory, Microbiological Diagnostic Unit, does not contain trimethoprim.

High rates of urine MCS testing in our study suggest that clinicians at our service rely on these assessments to assist in the diagnosis of a UTI and consider it best practice. This is contrary to Australian guidelines that advise that urine culture assessments may not be necessary for acute uncomplicated lower UTIs in nonpregnant adult females (9). These high rates could indicate clinician concerns about worsening antimicrobial resistance and also reflect the convenience of access to point-of-care testing at MSHC. As most patients in our study were tested for sexually transmitted infections (STIs) when they received a clinician-diagnosis of a UTI, diagnostic uncertainty may also have contributed. Given the potential overlap between UTI and STI presentations, conducting investigations for both in symptomatic females has been supported in some circumstances (26–28).

Empiric antibiotics were prescribed for >90% of episodes, with trimethoprim prescribed most often (>50% of prescriptions). Prescriptions almost always (96%) adhered to first-line empiric antibiotic recommendations (9). A 2020 Australian study that assessed prescribing practices in primary care found a similarly high proportion of prescriptions were provided empirically (86%) with high adherence to guideline recommendations (94%). Their study had comparable proportions of prescriptions for first-line antibiotics, with 50% of prescriptions for trimethoprim, and only 2% for nitrofurantoin (29). Despite being a first-line recommended antibiotic, nitrofurantoin was infrequently prescribed in our study; this may be due to its four times daily dosing requirement or adverse effect profile (9). Longer-acting nitrofurantoin formulations that allow for twice daily dosing are not registered in Australia (9, 30). Reducing the frequency of doses from four times daily to twice daily would likely reduce barriers to patient adherence, and encourage clinicians to prescribe nitrofurantoin.

Oral fosfomycin, administered as a single dose, is used as a first or second-line option in other countries and has now been introduced as a first-line option in Australia. We did not have data on fosfomycin susceptibility in our study as it was not included in the standard antimicrobial susceptibility testing panel. Fosfomycin was also not prescribed for any UTI episodes during our study period, reflecting its previous position in the Australian Therapeutic Guidelines as an alternative agent reserved for when there was resistance to other agents (9, 31). In a study assessing >1,000 *E. coli* UTI isolates from urine samples from Australian females 12 years of age or older in 2019, only two isolates were found to be resistant to fosfomycin (32). Thus, existing data suggest low rates of resistance to fosfomycin in Australian uropathogenic *E. coli,* supporting the Australian guidelines listing this agent as an option for empiric therapy.

### Limitations

A major limitation affecting most UTI studies is a poor ability to correctly identify all relevant uropathogens and confirm the cause of a UTI. For our study, a contributing factor to this limitation was that our clinic’s laboratory provider requires growth on culture of ≥10^3^ CFU/mL for a sample to be classified as a UTI. However, clinically-relevant growth can occur at lower colony counts of 10^1^–10^2^ CFU/mL in symptomatic females, which is lower than current standard microscopy or culture techniques can identify; therefore, some cases of UTI may have been missed by the laboratory reporting criteria of at least 10^3^ CFU/mL (33, 34). Furthermore, given increasing awareness that UTIs are more commonly polymicrobial than historically thought, traditional markers for contamination, including mixed growth samples, may result in misclassification of clinically significant culture findings (35). The large population size and retrospective nature of this study limited our ability to manually audit all records; hence, we could neither extract information on symptoms (including type and severity of symptoms), confirm that all included episodes were uncomplicated lower UTIs, or establish if a patient had a history of recurrent UTIs managed in other services. We assumed antibiotics prescribed before culture and susceptibility results were available were empiric; however, patients could have presented to another health service and had testing performed at an earlier date. As MSHC is the only public sexual health service for >6 million Victorians, patients are asked to see their general practitioner (GP) if their UTI symptoms do not resolve. Thus, we could not evaluate treatment outcomes for patients who received antibiotics or an antibiotic-sparing approach, or examine the proportion of patients recalled for a different antibiotic, as patients are typically called and advised to see their GP rather than return to MSHC. Our study included attendees at a large sexual health service who are typically young and sexually active, and therefore may not be generalizable to the general population. However, we focused on sexually active premenopausal nonpregnant persons with a vagina who are most impacted by uncomplicated lower UTIs. To our knowledge, this is the first study investigating uropathogen antimicrobial resistance at an Australian sexual health service. Our study contributes to the limited pre-existing Australian data on antimicrobial resistance in community-acquired UTIs, particularly in premenopausal, nonpregnant persons with a vagina.

### Conclusion

At Australia’s largest sexual health service, *E. coli* was responsible for two-thirds of diagnosed UTIs, and a third of *E. coli* isolates demonstrated trimethoprim resistance. This has important implications for clinical practice, as trimethoprim accounted for more than half of empiric antibiotic prescriptions, and trimethoprim resistance was present in isolate(s) from a quarter of episodes where it was prescribed. Overall, our findings suggest that changes to empiric antibiotic recommendations for acute uncomplicated lower UTIs in nonpregnant persons with a vagina may be needed. To ensure appropriate empiric antibiotic use, regular surveillance of local isolates and susceptibility profiles is recommended to inform treatment guidelines.

## MATERIALS AND METHODS

We undertook a retrospective analysis of premenopausal nonpregnant persons with a vagina diagnosed with a UTI at MSHC from 2 January 2018 to 24 January 2023.

### Inclusion criteria

Nonpregnant persons with a vagina who were premenopausal (defined as ≤51 years old [36]), had received a clinician-documented diagnosis of “urinary tract infection,” and had midstream urine sample results for urine MCS testing were eligible for inclusion.

We defined an acute UTI episode as the first presentation for a UTI at MSHC per 6-month period. For participants with multiple visits, only the first visit in the same 6-month period was included to prevent repeat presentations for the same UTI episode from influencing estimates. We excluded cisgender men and other adults who do not have a vagina, and patients who were pregnant, had pyelonephritis, or a renal transplant.

### Investigations

Most individuals presenting with urinary symptoms provide a midstream urine sample for urine dipstick and MCS testing, and testing for STIs is performed if clinically indicated. Urine dipstick (Siemens Multistix 10 SG) and microscopy testing (phase contrast microscopy on 400× magnification) are performed as point-of-care tests, with results available to clinicians during the patient’s initial presentation. The MSHC’s public health reference laboratory, the Microbiological Diagnostic Unit, defines significant levels of polymorphonuclear cells as ≥10 polymorphonuclear cells/L and bacteria as ≥1 bacterial cell per 10 small grids. While all included participants had samples sent for urine microscopy testing, culture was only performed if samples meet ≥1 of the following: positive nitrites or leukocyte esterase on urine dipstick, or >10 × 10^6^ polymorphonuclear cells/L on microscopy, or clinician request. The laboratory then classifies samples based on whether there is supporting microbiological evidence for “probable contamination,” “probable UTI,” or “possible UTI.” This classification is based on the degree of growth (i.e., number of colonies and CFU/mL), type of growth (e.g., pure or mixed) on culture, identification of a uropathogen, and presence of white blood cells and squamous epithelial cells on microscopy (see Supporting Information for full criteria). Antimicrobial susceptibility testing is only performed for laboratory-defined probable or possible UTI samples. Our laboratory performs sensitivities based on EUCAST guidelines on expected resistant and susceptible phenotypes. The antimicrobial resistance profiles of isolates were detected using the VITEK 2 instrument. VITEK results were interpreted according to European Committee on Antimicrobial Susceptibility Testing (EUCAST) clinical breakpoint criteria (relevant for the year of collection). Microbial tests of cure are not routinely recommended in national guidelines nor undertaken at MSHC.

### Data collection

Epidemiological (including gender and sexual practices), microbiological, and prescription data for eligible patients were extracted from three databases: (i) the MSHC’s electronic record system, Clinical Patient Management System; (ii) the MSHC’s laboratory provider, Microbiological Diagnostic Unit; and (iii) the MSHC’s prescription management software, Medical Director. Data regarding symptomatology was not available.

### Statistical analysis

For each acute episode of a clinician-diagnosed UTI, we calculated the following proportions with 95% confidence intervals (CI) using the binomial exact method: (i) proportion with specific findings on urine dipstick and microscopy, including nitrites, leukocyte esterase, and significant levels of polymorphonuclear cells and bacteria; (ii) proportion of culture samples with a uropathogen(s) isolated in laboratory-defined UTI episodes and, if available, their antimicrobial susceptibility profile; (iii) proportion of episodes in which an empiric antibiotic was prescribed, and if so, which antibiotic; (iv) proportion of empiric prescriptions that were compliant with current Australian Therapeutic Guideline recommendations for the empiric management of acute uncomplicated lower UTIs in nonpregnant adult females; and (v) proportion of episodes where the cultured isolate was resistant to the prescribed empiric antibiotic for that episode (9). All analyses were performed using STATA (version 17; StataCorp, College Station, USA).

Isolates reported by Microbiological Diagnostic Unit as “intermediate” or “susceptible, increased exposure” were classified as susceptible in the analyses (note that the EUCAST interpretation terminology changed from “intermediate” to “susceptible, increased exposure” during our study period; however, the minimum inhibitory concentrations did not change). Antibiotic prescriptions were classified as empiric if they were prescribed before urine culture and susceptibility results were available.

## Data Availability

The data for this study will not be shared as we do not have permission from the participants or ethics approval to do so. All authors had full access to all of the data related to the study.

## SUPPLEMENTAL MATERIAL

Supplemental material spectrum.02674-25-s0001.docx Interpretation of culture results, classification of urine culture growth, Tables A1 and A2, and Fig. A1.

ASM does not own the copyrights to Supplemental Material that may be linked to, or accessed through, an article. The authors have granted ASM a non-exclusive, world-wide license to publish the Supplemental Material files. Please contact the corresponding author directly for reuse.
