# Supplementary material for: Urinary tract infections at an Australian sexual health service: bacterial etiologies, antibiotic susceptibilities, and antimicrobial prescribing patterns
Source: Microbiol Spectr. 2026 May 5;14(6):e02674-25. doi: 10.1128/spectrum.02674-25 (PMC13228059; doi:10.1128/spectrum.02674-25)
Supplement: Supplemental material — Interpretation of culture results, classification of urine culture growth, Tables A1 and A2, and Fig. A1. [file spectrum.02674-25-s0001.docx]

# Supporting Information

## 1. Interpretation of culture results

The criteria by which Melbourne Sexual Health Centre’s (MSHC) laboratory, Melbourne Diagnostic Unit (MDU), classifies growth on urine culture is outlined below. Classifications assist in determining the clinical relevance of culture findings.

### Extent of growth

|  | **Number of colonies in total per equivalent CFU/ml** |
| --- | --- |
| **Heavy growth** | >100 colonies  >10^5^ CFU/ml |
| **Moderate growth** | 10-100 colonies  10^4^-10^5^ CFU/ml |
| **Light growth** | 1-10 colonies  10^3^-10^4^ CFU/ml |

CFU = colony forming unit

### Classification of urine culture growth

### Probable UTI classification

1. Heavy, pure growth of one isolate and >10x10^6^ white blood cells (WBCs)/L present

### Possible UTI classification

1. Heavy, pure growth of one isolate (WBCs absent)
2. Heavy growth of two isolates, each organism ≥100 colonies or between ≥10 and ≥100 colonies, and >10x10^6^ WBC/L present
3. Heavy growth of two or more isolates with one organism predominant, and >10x10^6^ WBC/L present or absent
4. Moderate, pure growth of one isolate, and >10x10^6^ WBC/L present
5. Moderate growth of two or more isolates with one organism predominant, with >10x10^6^ WBC/L present
6. Light, pure growth, and >10x10^6^ WBC/L present
7. Light growth of two organisms with similar amounts of growth (if both are possible pathogens, e.g. *E. coli* or *S. saprophyticus*), and >10x10^6^ WBC/L present

###

### Probable contamination classification

1. Moderate growth of two isolates, with growth of one pathogen at <10-100 colonies (but not predominant), and 10x10^6^ WBC/L absent +/- squamous epithelial cell(s)
2. Light mixed growth of three or more isolates +/- 10x10^6^ WBC/L +/- squamous epithelial cell(s)

##

## 2. Supplementary data

**Supplementary Data Table A1.** Co-infections by specimen type detected in participants at time of receiving a clinician-diagnosis of a urinary tract infection at Melbourne Sexual Health Centre between January 2018-January 2023.

|  | | **Number of episodes (any sample) (%)**  **n=1680** | **Number based on sample type (%)** | | | |
| --- | --- | --- | --- | --- | --- | --- |
|  |  |  | **First pass urine** | **High vaginal** | **Cervical** | **Rectal** |
| ***Neisseria gonorrhoeae*** | | | | | | |
|  | Detected | 33 (2.0) | 5 (0.3) | 19 (1.1) | 10 (0.6) | 3 (0.2) |
|  | Not detected | 1442 (85.8) | 450 (26.8) | 802 (47.7) | 211 (12.6) | 36 (2.1) |
|  | No data^a^ | 205 (12.2) | 1225 (72.9) | 859 (51.1) | 1459 (86.8) | 1641 (97.7) |
| ***Chlamydia trachomatis*** | | | | | | |
|  | Detected | 100 (6.0) | 37 (2.2) | 48 (2.9) | 15 (0.9) | 3 (0.2) |
|  | Not detected | 1379 (82.0) | 419 (24.9) | 775 (46.1) | 207 (12.3) | 36 (2.1) |
|  | No data^a^ | 201 (12.0) | 1224 (72.9) | 857 (51.0) | 1458 (86.8) | 1641 (97.7) |
| ***Mycoplasma genitalium*** | | | | | | |
|  | Detected | 27 (1.6) | 6 (0.4) | 18 (1.1) | 3 (0.2) | - |
|  | Not detected | 192 (11.4) | 51 (3.0) | 113 (6.7) | 29 (1.7) | - |
|  | No data^a^ | 1461 (87.0) | 1623 (96.6) | 1549 (92.2) | 1648 (98.1) | 1680 (100.0) |

^a^ No data includes participants who were not tested, and those whose results were classified as indeterminate or invalid on transcription-mediated amplification testing

**Supplementary Data Table A2.** Cultured isolates in laboratory-defined UTI samples (n=911) from patients attending Melbourne Sexual Health Centre from January 2018-January 2023.

| Isolate | Frequency, % [95%CI]  n=948 |
| --- | --- |
| *Escherichia coli* | 615, 64.9 [61.7-67.9] |
| *Staphylococcus saprophyticus* | 174, 18.4 [15.9-21.0] |
| *Klebsiella pneumoniae* | 43, 4.5 [3.3-6.1] |
| *Streptococcus agalactiae* | 39, 4.1 [2.9-5.6] |
| *Proteus mirabilis* | 20, 2.1 [1.3-3.2] |
| *Enterococcus faecalis* | 15, 1.6 [0.9-2.6] |
| *Citrobacter koseri* | 10, 1.1 [0.5-1.9] |
| *Klebsiella aerogenes* | 7, 0.7 [0.3-1.5] |
| *Staphylococcus aureus* | 7, 0.7 [0.3-1.5] |
| *Enterobacter cloaecae complex* | 4, 0.4 [0.1-1.1] |
| *Streptococcus pyogenes* | 3, 0.3 [0.1-0.9] |
| *Enterobacter aerogenes* | 2, 0.2 [0.0-0.8] |
| *Streptococcus dysgalactiae* | 2, 0.2 [0.0-0.8] |
| *Staphylococcus lugdunensis* | 2, 0.2 [0.0-0.8] |
| *Corynebacterium aurimucosium* | 1, 0.1 [0.0-0.6] |
| *Cronobacter malonaticus* | 1, 0.1 [0.0-0.6] |
| *Klebsiella oxytoca* | 1, 0.1 [0.0-0.6] |
| *Morganella morganii* | 1, 0.1 [0.0-0.6] |
| *Serratia marcescens* | 1, 0.1 [0.0-0.6] |

CI = confidence interval

NB For urine samples with >1 isolate, culture results for all isolates were included in this analysis

**
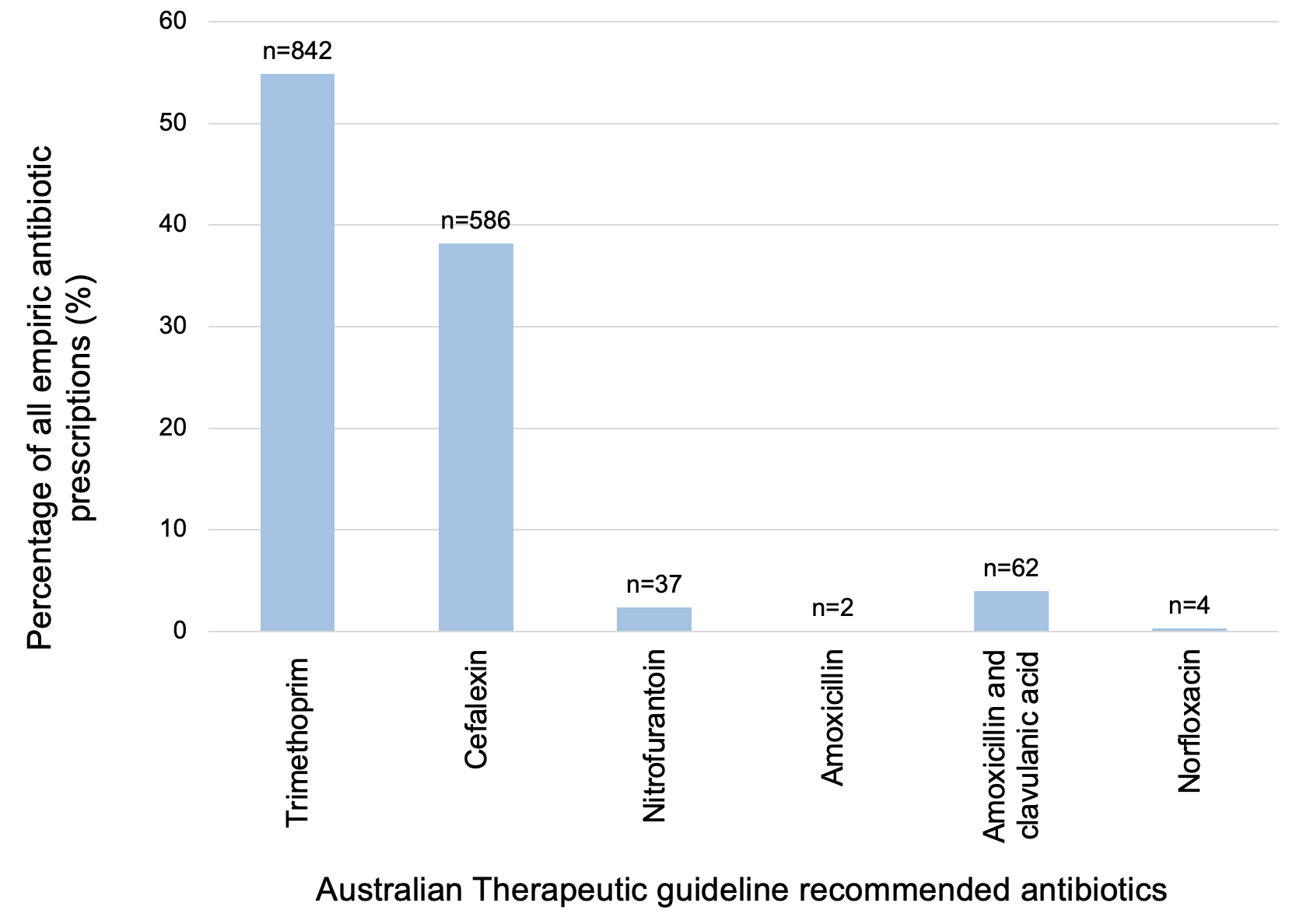
****Supplementary Data Figure A1. Empiric antibiotic prescriptions^a,b^ for clinician-diagnosed acute urinary tract infection (UTI) episodes at Melbourne Sexual Health Centre (MSHC) between January 2018-January 2023 (n=1533 prescriptions; n=1428 participants).**

^a^ There were 25/62 episodes where amoxicillin and clavulanic acid, and 1/2 episodes where amoxicillin was prescribed for patients with concurrent infections that influenced the prescribing practices of the clinician for their UTI – i.e. the clinician opted for a broader spectrum antibiotic to cover a concurrent diagnosis (e.g. pelvic inflammatory disease, bacterial vaginosis)

^b^ There were 13/62 episodes where amoxicillin and clavulanic acid, and 1/4 episodes where norfloxacin was prescribed for patients who had received recent antimicrobials from an external health service before their first presentation for that same UTI episode at MSHC
